# Supplementary material for: Oncogenic mutations of PIK3CA lead to increased membrane recruitment driven by reorientation of the ABD, p85 and C-terminus
Source: Nat Commun. 2023 Jan 12;14:181. doi: 10.1038/s41467-023-35789-6 (PMC9837058; doi:10.1038/s41467-023-35789-6)
Supplement: Supplementary file 1 — Supplementary Information [file 41467_2023_35789_MOESM1_ESM.pdf]

Supplementary Information for

Oncogenic mutations of *PIK3CA* lead to increased membrane recruitment driven by reorientation of the ABD, p85 and C-terminus

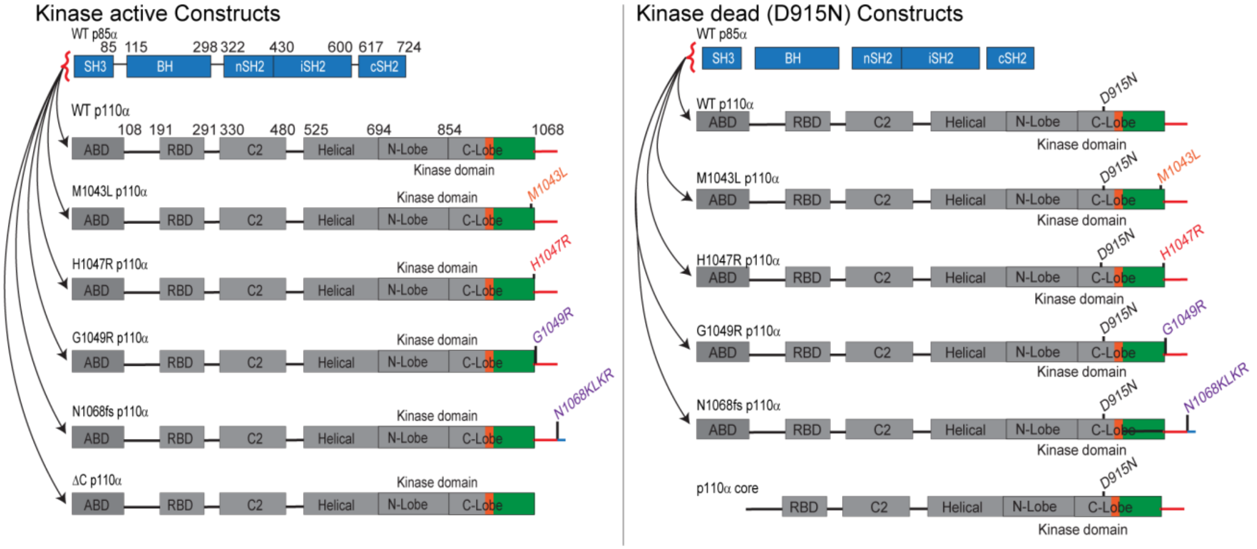

Supplementary Fig. 1- List of p110α and p85α constructs used.

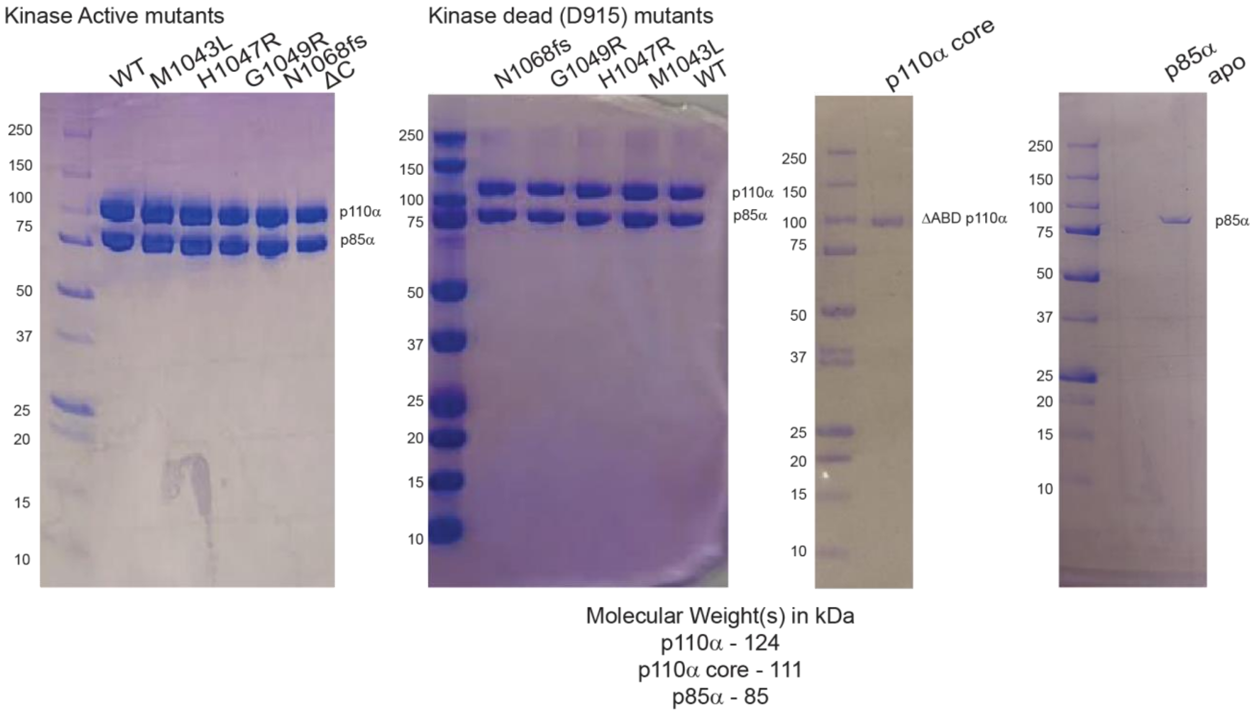

Supplementary Fig. 2. SDS PAGE gel images of all p110α constructs used in this study. These images (n=1) show the purity of each protein construct from a representative prep of each protein. Uncropped gel images are presented in the source data.

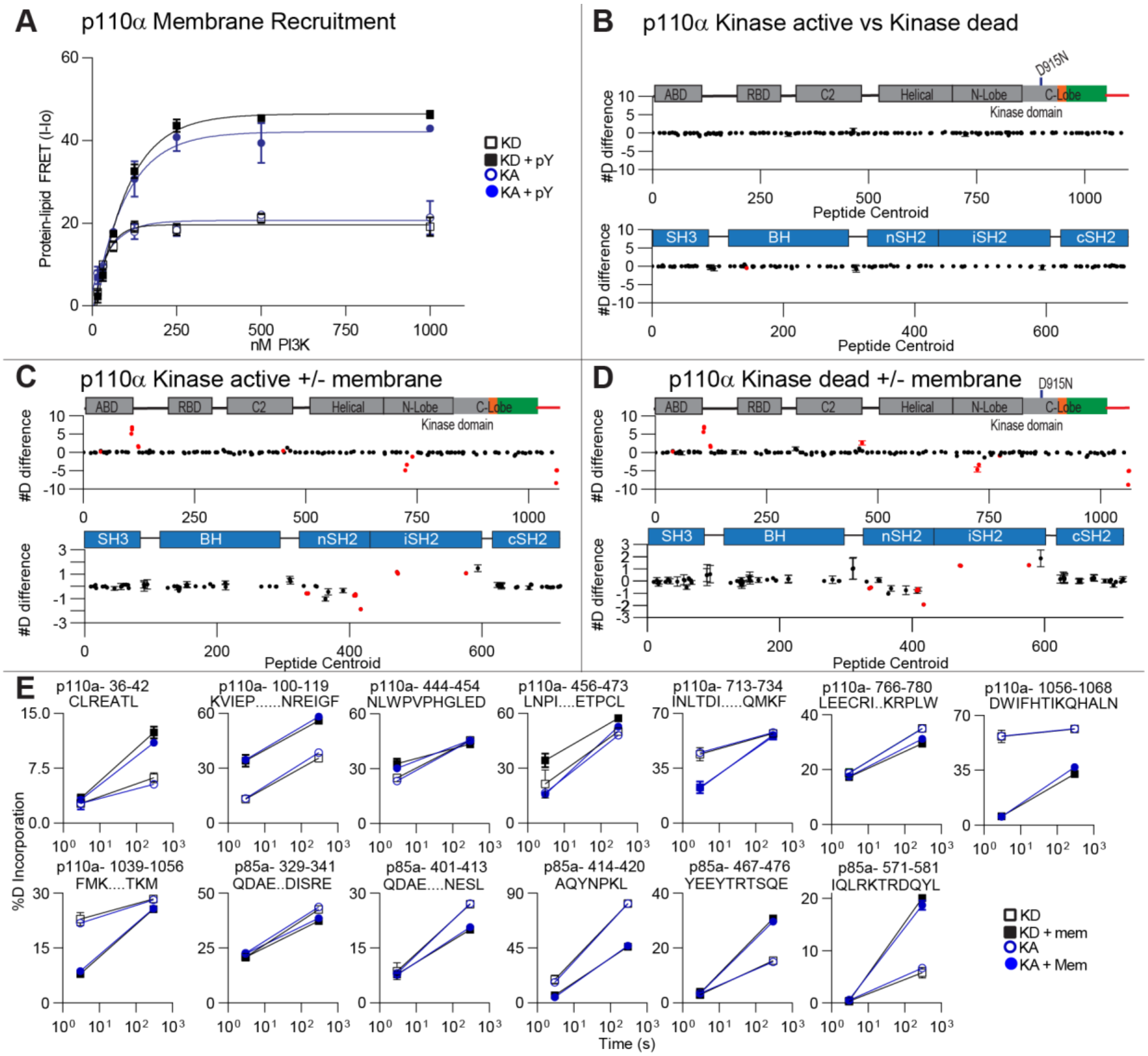

Supplementary Fig. 3 Comparison between kinase active (KA) and kinase dead (KD) p110α

A. Protein-Lipid FRET assay performed with kinase active and kinase dead p110α constructs under basal and pY activated states on liposomes containing 5% PIP_2, 10% Dansyl PS, 25 % PS, 65% PE. Experiments were carried out at saturating concentrations of PI3K concentrations ranging from 0.015 to 1µM, 1µM pY, and 16.65 µg/ml of lipid vesicles (technical replicates, error bars are S.D., n=3).

(B-D). The #D difference in deuterium incorporation for p110α and p85α in each experiment, with each point representing a single peptide, with error shown as the sum of SD across all time points (n=3 for each time point). comparing the following conditions: **C.** p110α/p85α WT kinase active vs kinase dead. **D.** WT kinase active in solution and membrane. **E.** WT kinase dead in solution and membrane. Peptides in p110α and p85α that showed significant differences in HDX upon binding to 100 nm extruded 5% PIP_2/PS/PE

vesicles are colored in red (greater than 0.4 Da and 5% difference at any timepoint, with a two tailed t-test $p<0.01$).

E. %D graphs for a selection of peptides comparing WT kinase active vs Kinase dead under pY active state and membrane bound state. (Mean is shown, with error bars representing S.D., n =3).. Full HDX-MS data available in source data.

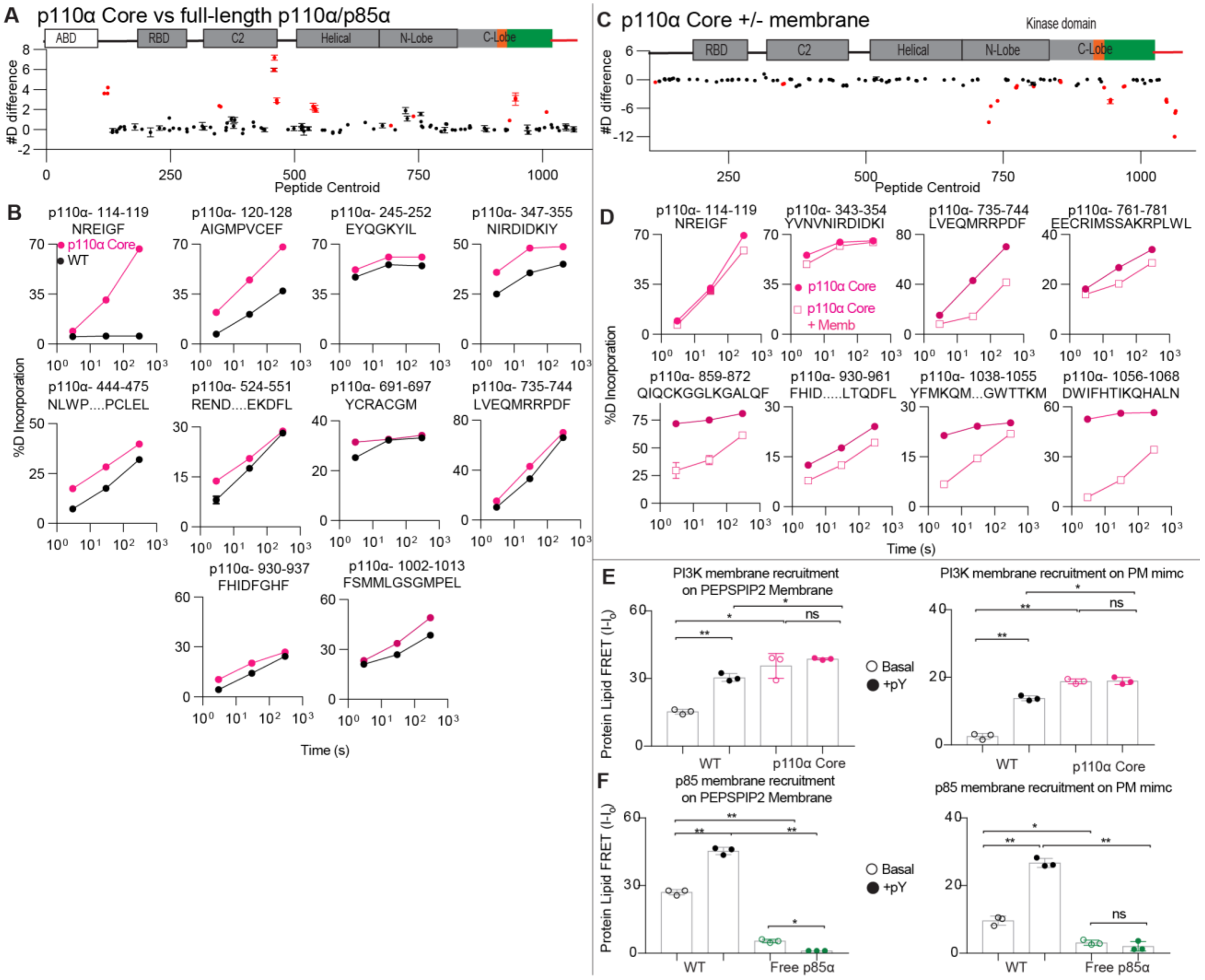

Supplementary Fig. 4 HDX-MS differences for experiments done with ΔABD

A. The #D difference in deuterium incorporation for p110α between p110α core and full-length p110α/p85α complex, each point represents a single peptide with error shown as the sum of SD across all time points (n=3 for each time point). Peptides in p110α that showed significant differences in HDX are colored in red (greater than 0.4 Da and 5% difference at any timepoint, with a two tailed t-test p<0.01).

B. %D graphs for a selection of peptides comparing p110α core and full-length p110α/p85α (Mean is shown, with error bars representing S.D., n =3). Full HDX-MS data available in source data.

C. The sum #D difference in deuterium incorporation across all time points for p110α core upon binding 5% PIP_2 membranes with error as SD (n=3 for each time point). Peptides in p110α that showed significant differences in HDX are colored in red (greater than 0.4 Da and 5% difference at any timepoint, with a two tailed t-test p<0.01).

D. %D graphs for a selection of peptides comparing for p110α core in the presence and absence of 5% PIP_2 membranes (Mean is shown, with error bars representing S.D., n =3). Note that the intrinsic exchange rate of different regions explains some of the differences in H/D exchange seen upon membrane binding. Regions with stable secondary structure in the absence of membrane are protected primarily at later time point (this is due to membrane binding further stabilising the secondary structure, see 735-744). The C-terminus undergoes a putative disorder-order transition (1056-1068), and shows stabilisation at all time points, with rapid exchange in the absence of membranes. Finally, regions with limited secondary structure (343-354) show protection at only early timepoints of D_2O exchange.

E. Protein-Lipid FRET testing membrane recruitment of p110α on PIP_2/PS/PE and PM mimic membranes. FRET assays were performed with saturating concentrations of PI3K (0.5 µM) and 16.65 µg of lipid (error bars are S.D., n=3). Two tailed p-values represented by the symbols as follows: **<0.001; *<0.05; n.s.>0.05.

F. Protein-Lipid FRET testing membrane recruitment of p85α on PIP_2/PS/PE and PM mimic membranes. FRET assays were performed with saturating concentrations of p85 (0.5 µM) and 16.65 µg of lipid (error bars are S.D., n=3). Two tailed p-values represented by the symbols as follows: **<0.001; *<0.05; n.s.>0.05. Source data for this figure are provided in the Source Data file.

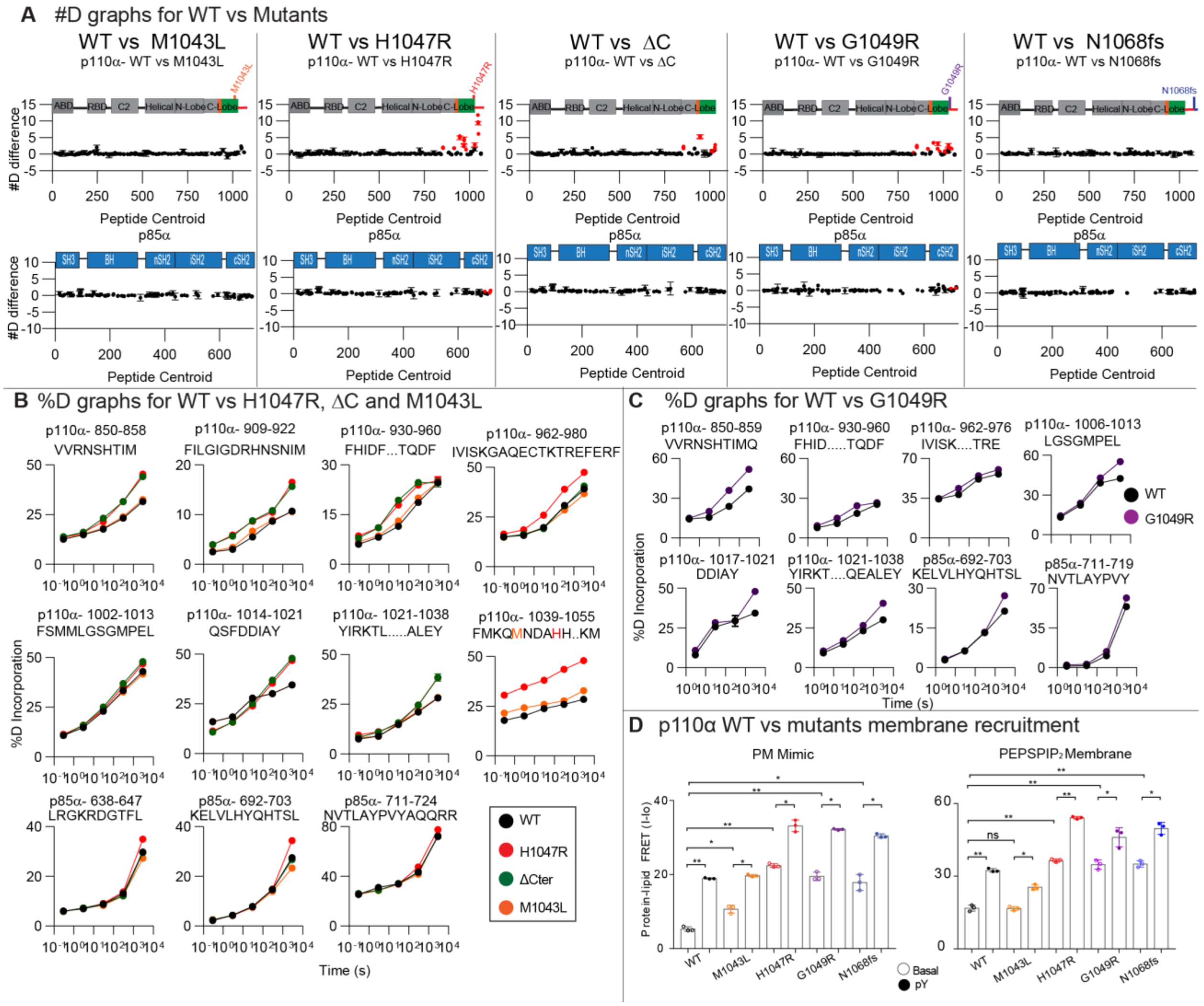

Supplementary Fig. 5 Source data for experiments comparing WT and different c-terminal mutants

A. The sum of the #D difference in deuterium incorporation across all time points for p110α and p85α in between WT and the indicated p110α mutant or deletion, each point represents a single peptide with error shown as the sum of SD across all time points (n=3 for each time point). Peptides in p110α and p85α that showed significant differences in HDX are colored in red (greater than 0.4 Da and 5% difference at any timepoint, with a two tailed t-test p<0.01). Peptide graphs for HDX differences between WT and mutants showing changes in both p110α and p85α **B-C**. % D graphs for a selection of peptides showing significant differences between and WT and mutants (Mean is shown, with error bars representing S.D., n =3). Full HDX-MS data available in the source data.

D. Protein-Lipid FRET assay performed with different p110α constructs under basal and pY activated states on PM mimic liposomes (5% PIP_2, 10% Dansyl PS, 15 % PS, 40% PE, 10% cholesterol, 15% PC and 5% SM) and 5% PIP_2, 10% Dansyl PS, 25 % PS, 65% PE liposomes. Experiments were carried out at saturating concentrations of PI3K (1µM) and 16.65 µg/ml of lipid vesicles (Mean is shown, with error bars representing S.D., n =3). Two tailed p-values represented by the symbols as follows: **<0.001; *<0.05; n.s.>0.05. Source data for this figure are provided in the Source Data file.

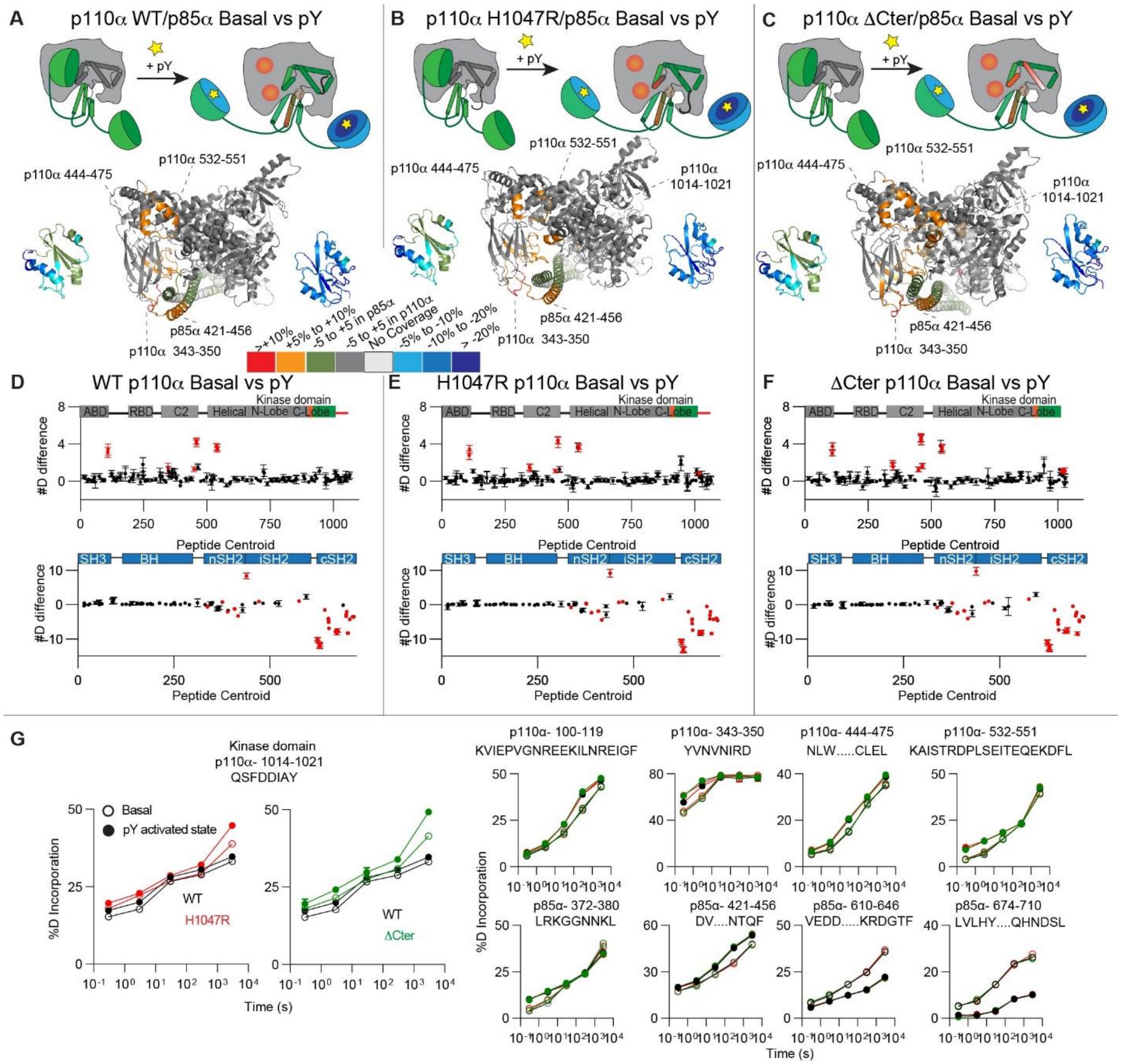

Supplementary Fig. 6. Addition of pY leads to increased exposure in the kinase domain.

A-C. HDX-MS comparing basal and pY activated states of (**A**) WT (**B**) H1047R and (**C**) the ΔC-terminus. The differences with H1047R were mapped onto p110α/iSH2-nSh2 (PDB: 3HHM) and cSH2 (2Y3A). The differences between WT and ΔCter were mapped onto p110α/iSH2-nSH2 (PDB: 4OVU) and nSH2 (2Y3A)

D-F. The sum of the #D difference in deuterium incorporation across all time points for p110α and p85α upon pY binding for the indicated p110α mutant or deletion, each point represents a single peptide with error shown as the sum of SD across all time points (n=3 for each time point). Peptides in p110α and p85α

that showed significant differences in HDX are colored in red (greater than 0.4 Da and 5% difference at any timepoint, with a two tailed t-test $p<0.01$).

G. %D graphs for a selection of peptides with differences in exchange, with all HDX-MS data available in the source data. Error bars are SD (n=3). Note that the intrinsic exchange rate of different regions explains some of the differences in H/D exchange seen upon pY binding. Regions with stable secondary structure in the absence of pY are protected primarily at later time points (this is due to pY binding further stabilising the secondary structure, see 1014-1021). Regions with less stable secondary structure show changes throughout the time course (see peptide 444-475). Source data for this figure are provided in the Source Data file.

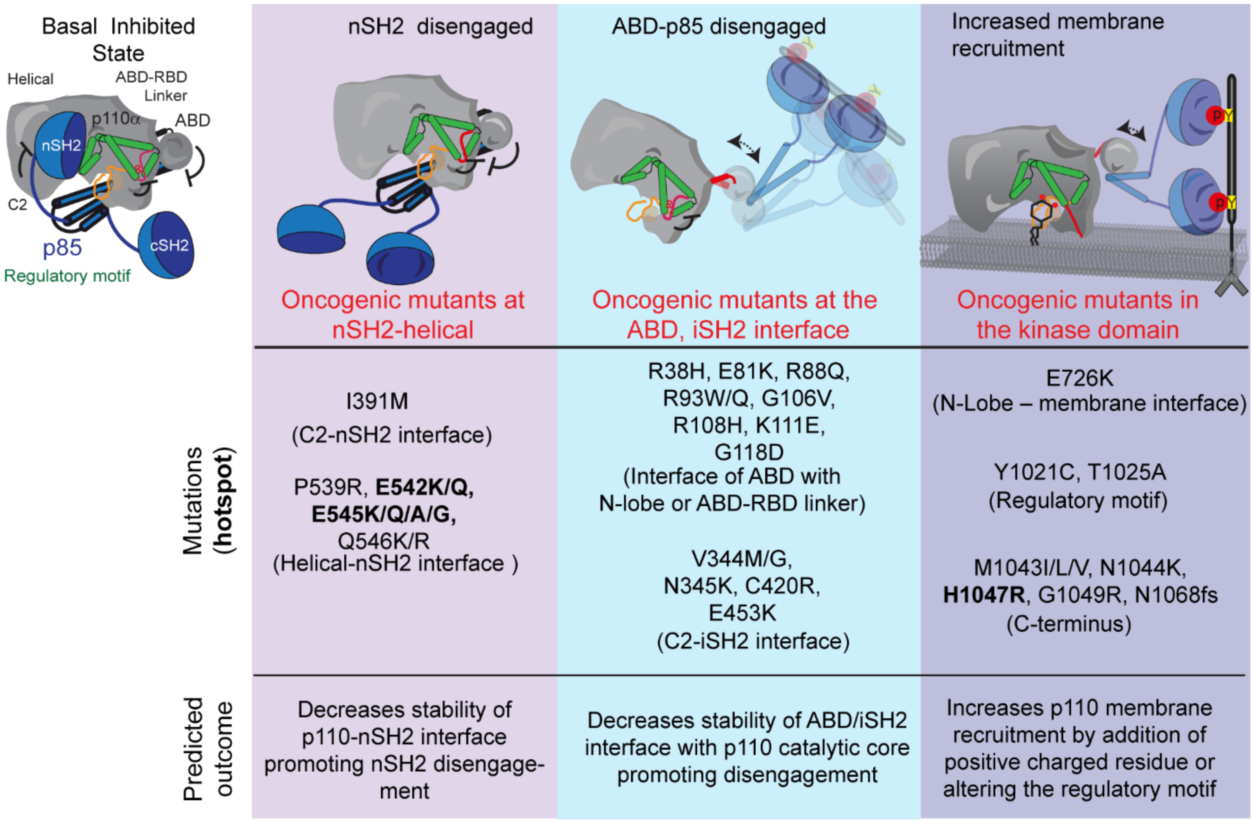

Supplementary Fig. 7: Predicted molecular mechanism of activation for various PIK3CA mutations. Mutations at the nSH2 interface/helical domain lead to disengagement of the nSH2 while the mutations at the ABD/C2 promote disengagement of ABD-p85 from the catalytic core. Mutations at the kinase domain promote membrane recruitment either by addition of positively charged residue at the membrane binding interface (E726K) or alter the regulatory motif.

Supplementary Table 1a. - HDX-MS processing details

Experiment	Comparing WT PI3K and p110α core. HDX Stats table for data shown in Figure 2 and Figure 3.		
Data set	WT	p110α core	p110α core + Memb
HDX reaction details	%D_2O=69%<br>$pH_{(read)}$=7.5<br>Temp=18ºC	%D_2O=69%<br>$pH_{(read)}$=7.5<br>Temp=18ºC	%D_2O=69%<br>$pH_{(read)}$=7.5<br>Temp=18ºC
HDX time course (seconds)	3s, 30s, 300s	3s, 30s, 300s	3s, 30s, 300s
HDX controls	N/A	N/A	N/A
Back-exchange	No correction, deuterium levels are relative	No correction, deuterium levels are relative	No correction, deuterium levels are relative
Replicates	3	3	3
Protein	p110a		
Number of peptides	125	125	125
Sequence coverage	89.9%	89.9%	89.9%
Average peptide /redundancy	Length= 13.9<br>Redundancy= 1.8	Length= 13.9<br>Redundancy= 1.8	Length= 13.9<br>Redundancy= 1.8
Repeatability	Average StDev= 0.6%	Average StDev=0.4%	Average StDev=0.6%

Supplementary Table 1b- HDX-MS processing details

Experiment	Comparing basal PI3K WT vs mutants (H1047R, M1043L and Delta C) and in the presence of pY with H1047R and Delta C. HDX Stats table for data shown in Figure 5A, Figure 5C, Figure 5D and Figure S7						
Data set	WT	WT+pY	H1047R	H1047R + pY	M1043L	Delta C	Delta C + pY
HDX reaction details	%D_2O=75%<br>$pH_{(read)}$=7.5<br>Temp=18ºC	%D_2O=75%<br>$pH_{(read)}$=7.5<br>Temp=18ºC	%D_2O=75%<br>$pH_{(read)}$=7.5<br>Temp=18ºC	%D_2O=75%<br>$pH_{(read)}$=7.5<br>Temp=18ºC	%D_2O=75%<br>$pH_{(read)}$=7.5<br>Temp=18ºC	%D_2O=75%<br>$pH_{(read)}$=7.5<br>Temp=18ºC	%D_2O=75%<br>$pH_{(read)}$=7.5<br>Temp=18ºC
HDX time course (seconds)	3s, 30s, 300s, 3000s at 18ºC<br>3s at 0 ºC	3s, 30s, 300s, 3000s at 18ºC<br>3s at 0 ºC	3s, 30s, 300s, 3000s at 18ºC<br>3s at 0 ºC	3s, 30s, 300s, 3000s at 18ºC<br>3s at 0 ºC	3s, 30s, 300s, 3000s at 18ºC<br>3s at 0 ºC	3s, 30s, 300s, 3000s at 18ºC<br>3s at 0 ºC	3s, 30s, 300s, 3000s at 18ºC<br>3s at 0 ºC
HDX controls	N/A	N/A	N/A	N/A	N/A	N/A	N/A
Back-exchange	No correction deuterium levels are relative	No correction deuterium levels are relative	No correction deuterium levels are relative	No correction deuterium levels are relative	No correction deuterium levels are relative	No correction deuterium levels are relative	No correction deuterium levels are relative
Replicates	3	3	3	3	3	3	3 (2 for 3s 0 ºC at sample)
Significant differences in HDX	>5% and >0.4 Da and unpaired t-test ≤0.01	>5% and >0.4 Da and unpaired t-test ≤0.01	>5% and >0.4 Da and unpaired t-test ≤0.01	>5% and >0.4 Da and unpaired t-test ≤0.01	>5% and >0.4 Da and unpaired t-test ≤0.01	>5% and >0.4 Da and unpaired t-test ≤0.01	>5% and >0.4 Da and unpaired t-test ≤0.01
Protein							
Number peptides	171	171	170	170	169	164	164
Sequence coverage	95.8%	95.8%	95.8%	95.8%	95.8%	95.8%	95.8%
Average peptide /redundancy	Length= 13.7 Redundancy= 2.1	Length= 13.7 Redundancy= 2.1	Length= 13.7 Redundancy= 2.1	Length= 13.7 Redundancy= 2.1	Length= 13.7 Redundancy= 2.1	Length= 13.7 Redundancy= 2.1	Length= 13.7 Redundancy= 2.1
Repeatability	Average StDev=0.5%	Average StDev=0.5%	Average StDev=0.6%	Average StDev=0.5%	Average StDev=0.5%	Average StDev=0.5%	Average StDev=0.5%
Protein							
Number peptides	117	117	117	117	117	117	117
Sequence coverage	90.3%	90.3%	90.3%	90.3%	90.3%	90.3%	90.3%
Average peptide /redundancy	Length= 16.3 Redundancy= 2.6	Length= 16.3 Redundancy= 2.6	Length= 16.3 Redundancy= 2.6	Length= 16.3 Redundancy= 2.6	Length= 16.3 Redundancy= 2.6	Length= 16.3 Redundancy= 2.6	Length= 16.3 Redundancy= 2.6
Repeatability	Average StDev=0.5%	Average StDev=0.6%	Average StDev=0.6%	Average StDev=0.5%	Average StDev=0.6%	Average StDev=0.6%	Average StDev=0.6%

Supplementary Table 1c- HDX-MS processing details

Experiment	Comparing Kinase Active PI3K and G1049R PI3K. HDX Stats table for data shown in Figure 5B and Figure 5F	
Data set	WT	G1049R
HDX reaction details	%D_2O=76%<br>$pH_{(read)}$=7.5<br>Temp=18ºC	%D_2O=76%<br>$pH_{(read)}$=7.5<br>Temp=18ºC
HDX time course (seconds)	3s, 30s, 300s, 3000s	3s, 30s, 300s, 3000s
HDX controls	N/A	N/A
Back-exchange	No correction, deuterium levels are relative	No correction, deuterium levels are relative
Replicates	3	3
Significant differences in HDX	>5% and >0.4 Da and unpaired t-test ≤0.01	>5% and >0.4 Da and unpaired t-test ≤0.01
Protein	p110a	
Number of peptides	169	169
Sequence coverage	95.2%	95.2%
Average peptide /redundancy	Length=13.7<br>Redundancy=2.1	Length=13.7<br>Redundancy=2.1
Repeatability	Average StDev=0.6%	Average StDev=0.5%
Protein	p85	
Number of peptides	114	114
Sequence coverage	89%	89%
Average peptide /redundancy	Length=16.4<br>Redundancy=2.5	Length=16.4<br>Redundancy=2.5
Repeatability	Average StDev=0.6%	Average StDev=0.5%

Experiment	Comparing Kinase Active PI3K and Frameshift. HDX Stats table for data shown in Figure 5E	
Data set	WT	Frameshift
HDX reaction details	%D_2O=81%<br>$pH_{(read)}$=7.5<br>Temp=18ºC	%D_2O=81%<br>$pH_{(read)}$=7.5<br>Temp=18ºC
HDX time course (seconds)	3s, 30s, 300s, 3000s	3s, 30s, 300s, 3000s
HDX controls	N/A	N/A
Back-exchange	No correction, deuterium levels are relative	No correction, deuterium levels are relative
Replicates	3 (2 for 3s sample)	3
Significant differences in HDX	>5% and >0.4 Da and unpaired t-test ≤0.01	>5% and >0.4 Da and unpaired t-test ≤0.01
Protein	p110a	
Number of peptides	141	139
Sequence coverage	84.7%	84.7%
Average peptide /redundancy	Length= 13.7<br>Redundancy= 1.6	Length= 13.7<br>Redundancy= 1.6
Repeatability	Average StDev=0.9%	Average StDev=0.7%
Protein	p85	
Number of peptides	180	180
Sequence coverage	79%	79%
Average peptide /redundancy	Length= 16<br>Redundancy= 2.6	Length= 16<br>Redundancy= 2.6
Repeatability	Average StDev=0.9%	Average StDev=0.6%

Supplementary Table 1d-HDX-MS processing details

Experiment	Comparing Kinase Active PI3K and Kinase Dead (KD) PI3K. HDX Stats table for data shown in Supplemental Figure 3			
Data set	WT	WT+memb	KD	KD + Memb
HDX reaction details	%D_2O=72%<br>$pH_{(read)}$=7.5<br>Temp=18ºC	%D_2O=72%<br>$pH_{(read)}$=7.5<br>Temp=18ºC	%D_2O=72%<br>$pH_{(read)}$=7.5<br>Temp=18ºC	%D_2O=72%<br>$pH_{(read)}$=7.5<br>Temp=18ºC
HDX time course (seconds)	3s, 300s	3s, 300s	3s, 300s	3s, 300s
HDX controls	N/A	N/A	N/A	N/A
Back-exchange	No correction, deuterium levels are relative	No correction, deuterium levels are relative	No correction, deuterium levels are relative	No correction, deuterium levels are relative
Replicates	3	3	3	3
Significant differences in HDX	>5% and >0.4 Da and unpaired t-test ≤0.01	>5% and >0.4 Da and unpaired t-test ≤0.01	>5% and >0.4 Da and unpaired t-test ≤0.01	>5% and >0.4 Da and unpaired t-test ≤0.01
Protein	p110a			
Number of peptides	133	133	133	133
Sequence coverage	84.9%	84.9%	84.9%	84.9%
Average peptide /redundancy	Length=13.1<br>Redundancy=1.5	Length=13.1<br>Redundancy=1.5	Length=13.1<br>Redundancy=1.5	Length=13.1<br>Redundancy=1.5
Repeatability	Average StDev=0.7	Average StDev=0.7	Average StDev=1.1%	Average StDev=1.3%
Protein	p85			
Number of peptides	81	81	81	81
Sequence coverage	71.4%	71.4%	71.4%	71.4%
Average peptide /redundancy	Length=15.1<br>Redundancy=1.6	Length=15.1<br>Redundancy=1.6	Length=15.1<br>Redundancy=1.6	Length=15.1<br>Redundancy=1.6
Repeatability	Average StDev=0.7	Average StDev=0.6%	Average StDev=1.4%	Average StDev=1%
